# Biomechanical analysis of sandwich vertebrae in osteoporotic patients: finite element analysis

**DOI:** 10.3389/fendo.2023.1259095

**Published:** 2023-10-11

**Authors:** Shaolong Huang, Chengqiang Zhou, Xu Zhang, Zhongjian Tang, Liangyu Liu, Xiao Meng, Cheng Xue, Xianye Tang

**Affiliations:** ^1^ Department of Orthopedics, The Affiliated Hospital of Xuzhou Medical University, Xuzhou, Jiangsu, China; ^2^ Graduate School of Xuzhou Medical University, Xuzhou, Jiangsu, China; ^3^ North Sichuan Medical College, Nanchong, Sichuan, China

**Keywords:** sandwich vertebra, osteoporotic vertebral compression fracture, percutaneous vertebroplasty, adjacent vertebral fracture, finite element analysis

## Abstract

**Objective:**

The aim of this study was to investigate the biomechanical stress of sandwich vertebrae (SVs) and common adjacent vertebrae in different degrees of spinal mobility in daily life.

**Materials and methods:**

A finite element model of the spinal segment of T10-L2 was developed and validated. Simultaneously, T11 and L1 fractures were simulated, and a 6-ml bone cement was constructed in their center. Under the condition of applying a 500-N axial load to the upper surface of T10 and immobilizing the lower surface of L2, moments were applied to the upper surface of T10, T11, T12, L1, and L2 and divided into five groups: M-T10, M-T11, M-T12, M-L1, and M-L2. The maximum von Mises stress of T10, T12, and L2 in different groups was calculated and analyzed.

**Results:**

The maximum von Mises stress of T10 in the M-T10 group was 30.68 MPa, 36.13 MPa, 34.27 MPa, 33.43 MPa, 26.86 MPa, and 27.70 MPa greater than the maximum stress value of T10 in the other groups in six directions of load flexion, extension, left and right lateral bending, and left and right rotation, respectively. The T12 stress value in the M-T12 group was 29.62 MPa, 32.63 MPa, 30.03 MPa, 31.25 MPa, 26.38 MPa, and 26.25 MPa greater than the T12 stress value in the other groups in six directions. The maximum stress of L2 in M-T12 in the M-L2 group was 25.48 MPa, 36.38 MPa, 31.99 MPa, 31.07 MPa, 30.36 MPa, and 32.07 MPa, which was greater than the stress value of L2 in the other groups. When the load is on which vertebral body, it is subjected to the greatest stress.

**Conclusion:**

We found that SVs did not always experience the highest stress. The most stressed vertebrae vary with the degree of curvature of the spine. Patients should be encouraged to avoid the same spinal curvature posture for a long time in life and work or to wear a spinal brace for protection after surgery, which can avoid long-term overload on a specific spine and disrupt its blood supply, resulting in more severe loss of spinal quality and increasing the possibility of fractures.

## Introduction

Osteoporosis and osteoporotic vertebral compression fractures (OVCFs) are a growing concern due to the aging of the global population (1). Osteoporosis is a systemic metabolic disease characterized by osteopenia and susceptibility to fractures, and osteoporotic vertebral compression fractures are the most prevalent type of osteoporotic fracture (2, 3). These fractures can result in back pain, limited spinal function, loss of vertebral height, kyphotic deformity, lung and gastrointestinal problems, and a poor quality of life for the patients. The increasing incidence of OVCF imposes an increasing burden on society and has become a major global health problem (2).

Percutaneous vertebroplasty (PVP) is a minimally invasive procedure in which bone cement is injected percutaneously into the vertebral body. PVP can rapidly alleviate pain, restore vertebral body height, improve function and range of motion, and reduce mortality and the incidence of complications in OVCF patients (3, 4). However, it has been shown that patients with OVCF who receive PVP have an increased risk of subsequent vertebral fractures and a greater risk of fractures in adjacent vertebrae than in other vertebrae (3, 5). New adjacent vertebral fractures may be attributable to increased spine stiffness due to cement augmentation, altered load transfer, progression of osteoporosis, polymethyl methacrylate (PMMA) filling pattern, and cement volume (6, 7). Solitro et al. also focused on correlating the fracture load of the region subject to fracture to the load for the entire vertebrae (8). When multiple vertebral surgeries are performed on a single patient, a sandwich vertebra (SV) may be formed, which is an intact, unreinforced vertebra sandwiched between two cemented vertebral bodies. It has been suggested that sandwich vertebrae are subjected to double loading and hypothesized to be more susceptible to secondary fractures than other vertebrae, but only a few clinical articles have addressed sandwich vertebrae (1, 7, 9). Whether SV is a high-risk vertebra is only theorized, and whether SV should undergo preventive cement augmentation and other conclusions are inconsistent. No biomechanical research has been conducted on SV.

Therefore, we developed a sandwich vertebral model using the finite element (FE) analysis method to study and observe the biomechanical status of SV and upper and lower adjacent vertebrae during different activities by applying different moments to the model, providing a biomechanical basis for the guidance of postoperative nursing management treatment for patients with sandwich vertebrae in clinical practice and filling the gap in this area of research.

## Materials and methods

The software used in this study is Mimics 21.0 (Materialise, Leuven, Belgium), Geomagic 2021 (Geomagic, Research Triangle Park, NC, USA), SolidWorks 2021 (Dassault Systemes, Waltham, MA, USA), and ANSYS 19.0 (ANSYS, Canonsburg, PA, USA).

### The construction of the T10-L2 finite element model

Spinal CT data were obtained from a healthy male volunteer (70 kg, 175 cm) with no history of spinal trauma or osteoporosis. This study was reviewed by our hospital ethics committee, and the volunteer provided signed informed consent. With the use of DICOM format data files, CT-scanned spine data were imported into MIMICS software for three-dimensional model reconstruction. The reconstructed model was imported into Geomagic Studio 2021 software in STL format, processed backward into a 3D geometric model, and optimized for surface smoothing, cortical and cancellous bone separation, noise reduction, and surface fitting.

The 3D geometric model generated by Geomagic 2021 was exported as a stp format file and then imported into SolidWorks 2021 software to assemble the vertebral body. With the use of the placement convex table and segmentation function, the cortical and cancellous bones were trimmed and combined, and the lumbar disc, annulus fibrosus, nucleus pulposus, endplate, and articular cartilage were created. The following linear ligaments were added: anterior longitudinal ligament, posterior longitudinal ligament, interspinous ligament, supraspinous ligament, ligamentum flavum, intertransverse ligament, and capsular ligament. The cortical bone thickness of the vertebral body was set at 1.5 mm, and the endplate thickness was 0.5 mm (10, 11).

After the creation of the 3D model in SolidWorks 2021, it was imported into ANSYS Workbench 19.0 for tetrahedral meshing of the vertebrae, vertebral bodies, endplates, and intervertebral discs. The mesh size of articular cartilage was 0.5 mm, while that of all others was 2 mm (12, 13). The spine and intervertebral disc were analyzed as separate entities, with the element type being a tetrahedral 10-node element and the ligament being LINK180, which was only stretched.

### The simulation of vertebral fractures treated by PVP

The fracture line model was created in Materialise Magics 21.0 by cutting the vertebral body to create a 0.5-mm fracture line with the slit penetrating the vertebral body through the center of the anterior cortical shell and the depth, width, and height of the slit being approximately 22 mm, 47.5 mm, and 0.5 mm, respectively. The bone cement was made by creating a 6-ml cylinder in SolidWorks 2021 (14), placing the cylinder in the positive center of the T11 and L1 vertebral bodies and then performing a deletion combination. The mechanical properties of the cylinder are specified as linear elasticity, isotropy, and homogeneity, and the interface between the vertebral body and the cylinder is specified as fully bound.

### Experimental group

**Figure 1:**
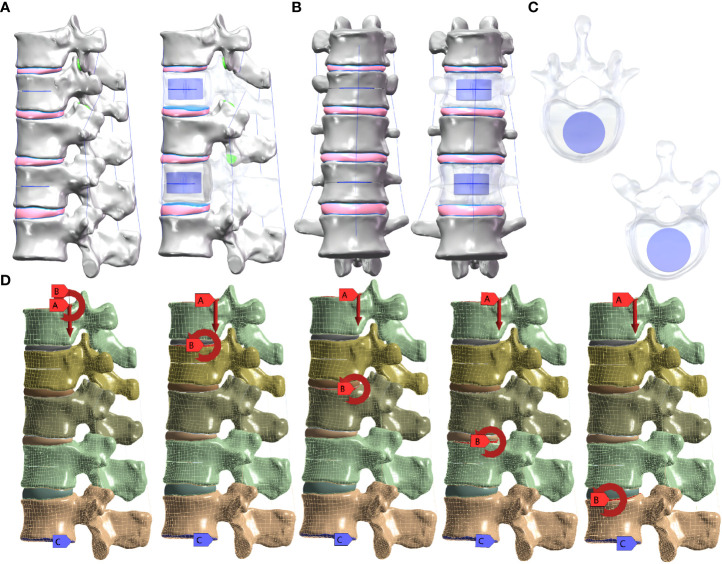
**(A)** Lateral view of the sandwich vertebra model. **(B)** Frontal view of the sandwich vertebra model. **(C)** Top view of T11 and L1. **(D)** Moment on the superior surface of each vertebra when a load of 500 N is applied to the superior surface of T10 and the inferior surface of L2 is fixed.

The weight of the upper segment of the human body was simulated by applying an axial compressive load of 500 N to the upper surface of the T10 vertebral body and setting pure moment of 7.5 N/m in six directions: forward flexion, extension, left/right bending, and left/right axial rotation were applied to the upper surface of the T10, T11, T12, L1, and L2 vertebral bodies, respectively, while the lower surface of the L2 vertebral body was fixed in all directions. The five groups of M-T10, M-T11, M-T12, M-L1, and M-L2 were formed (**Figure 1**).

### Boundary and loading conditions of FE models

According to previous research data, each material was assigned a value, and osteoporosis was simulated using 67% of the normal elastic modulus (**Table 1**) (11, 15–18). The facet joint contact was defined as surface–surface contact. The friction coefficient was set as 0.1, and the contact interface of other components was set as binding contact (19). For each group, the magnitude of the maximum von Mises stresses in the T10, T12, and L2 vertebrae was analyzed.

**Table 1:** Material properties information consists of finite elements models.

Parts	Young’s modulus (MPa)	Poisson ratio	Sectional area (mm^2^)
Normal cortical bone	12,000	0.3	–
Osteoporotic cortical bone	8,040 (67% of normal)	0.3	–
Normal cancellous bone	132	0.2	–
Osteoporotic cancellous bone	34 (67% of normal)	0.2	–
Normal endplate	1,000	0.4	–
Osteoporotic endplate	670 (67% of normal)	0.4	–
Cartilage	10	0.4	–
Annulus fibrosus	4.2	0.45	–
Nucleus pulposus	1	0.499	–
Bone cement	3,000	0.4	–
Anterior longitudinal ligament	20	0.3	65
Posterior longitudinal ligament	20	0.3	20
Ligamentum flavum	19.5	0.3	40
Supraspinous ligament	15	0.3	30
Interspinous ligament	12	0.3	40
Intertransverse ligament	59	0.3	1.8
Capsular ligament	7.5	0.3	30

## Results

### Validation of the intact mode

**Figure 2:**
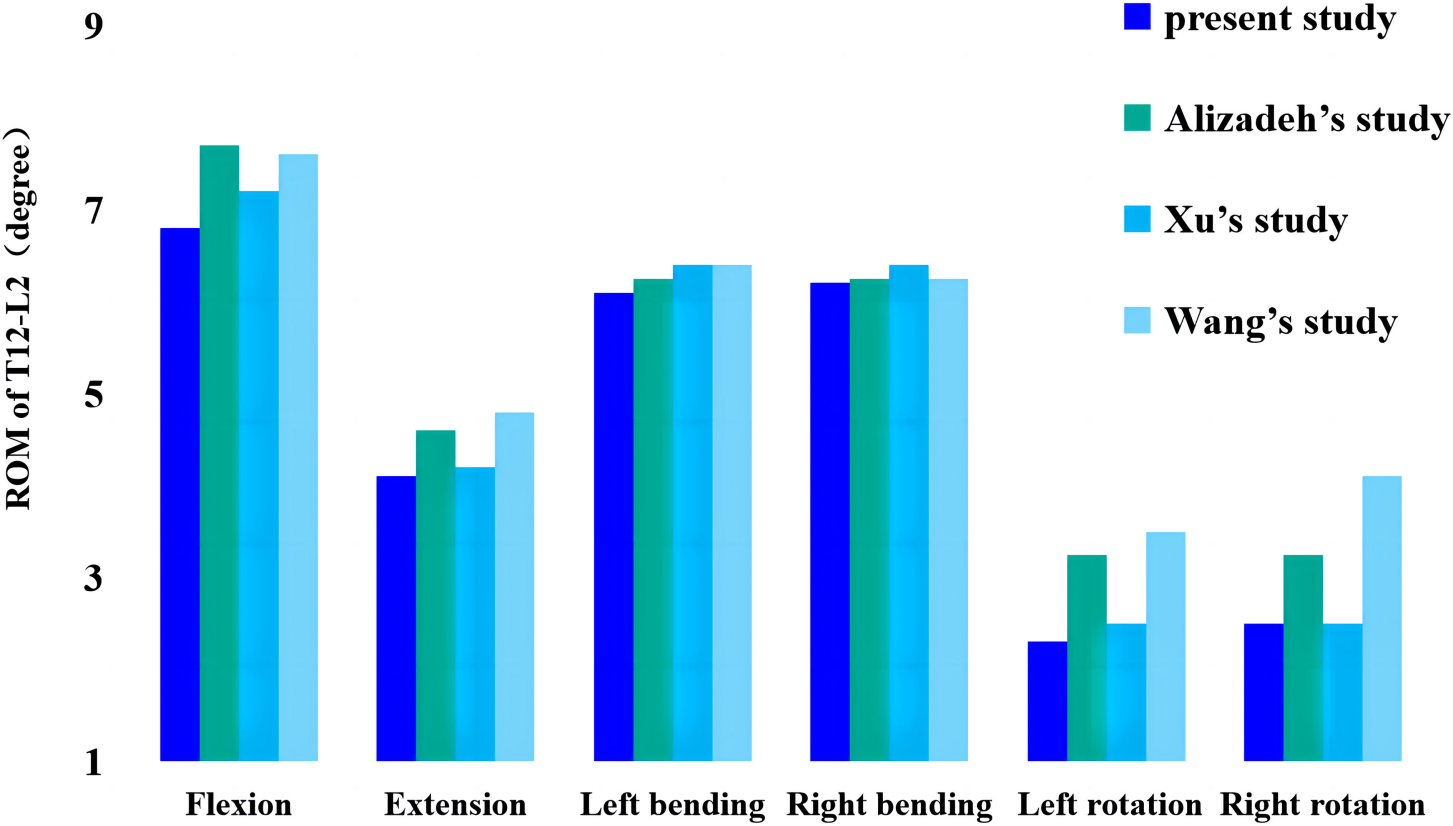
Comparison of this study’s ROM values from thoracolumbar models with previously reported values. ROM, range of motion.

**Figure 2** shows the comparison of the range of motion (ROM) values obtained in this study for the T12-L2 level and previously published biomechanical and finite element analysis data measuring flexion, extension, lateral bending, and axial rotation. The model-predicted ROM of the T12-L2 connection is consistent with experimental data from previous studies, validating the current thoracolumbar model for use in future studies (20–22).

### Maximum von Mises stress values for T10, T12, and L2 in M-T10

**Figure 3:**
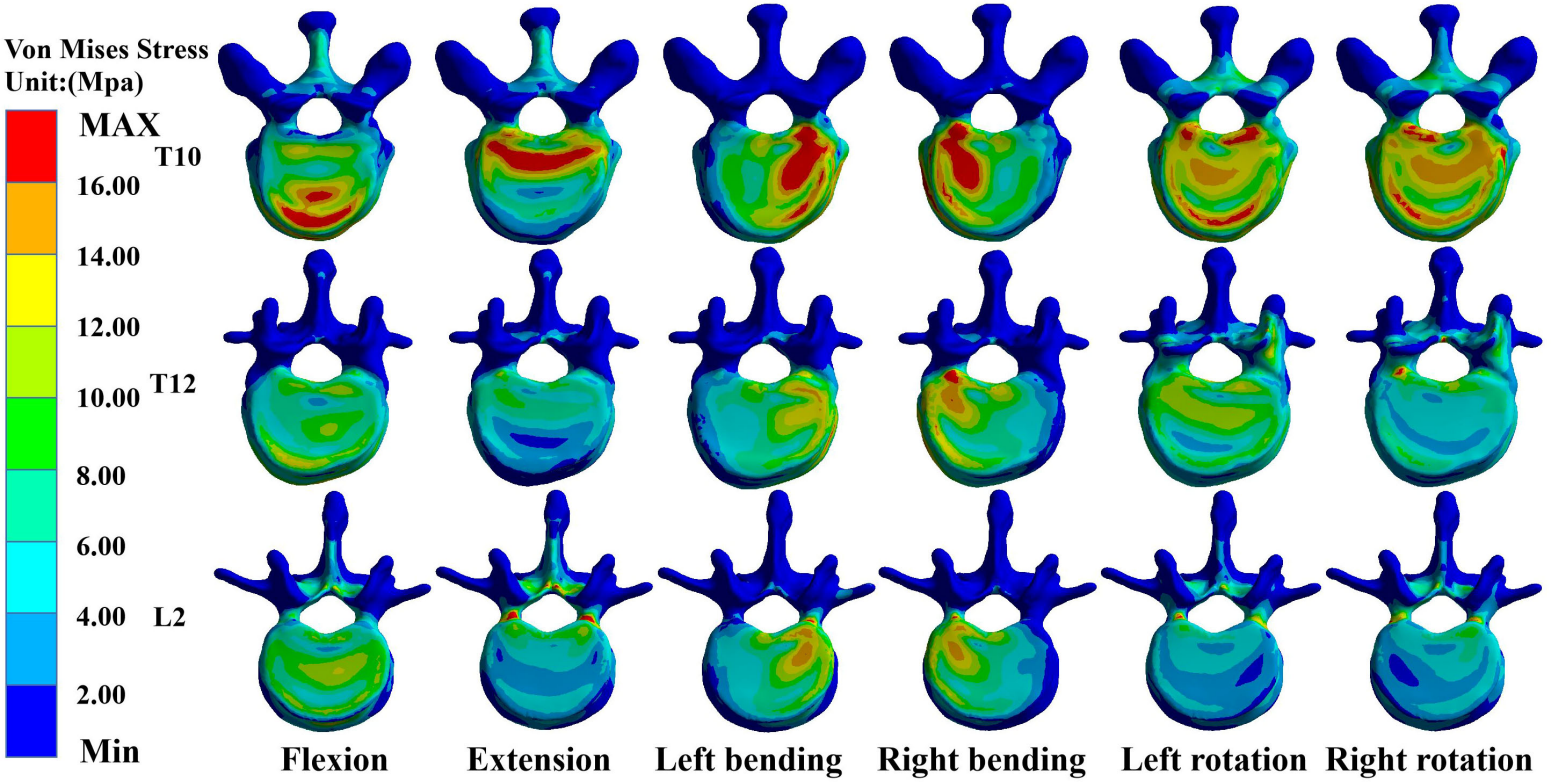
Maximum von Mises stress at T10, T12, and L2 in M-T10 group.

In the M-T10, T10 showed the largest von Mises stress value. For T10, the maximum von Mises stress values were 30.68 MPa, 36.13 MPa, 34.27 MPa, 33.43 MPa, 26.86 MPa, and 27.71 MPa in the six directions of forward flexion, extension, left and right lateral bending, and left and right rotation, respectively; those for T12 were 21.741 MPa, 18.76 MPa, 25.48 MPa, 27.41 MPa, 20.87 MPa, and 20.53 MPa in the six directions; and those for L2 were 20.83 MPa, 30.61 MPa, 26.15 MPa, 24.15 MPa, 19.54 MPa, and 17.54 MPa (**Figure 3**).

### Maximum von Mises stress values for T10, T12, and L2 in M-T11

**Figure 4:**
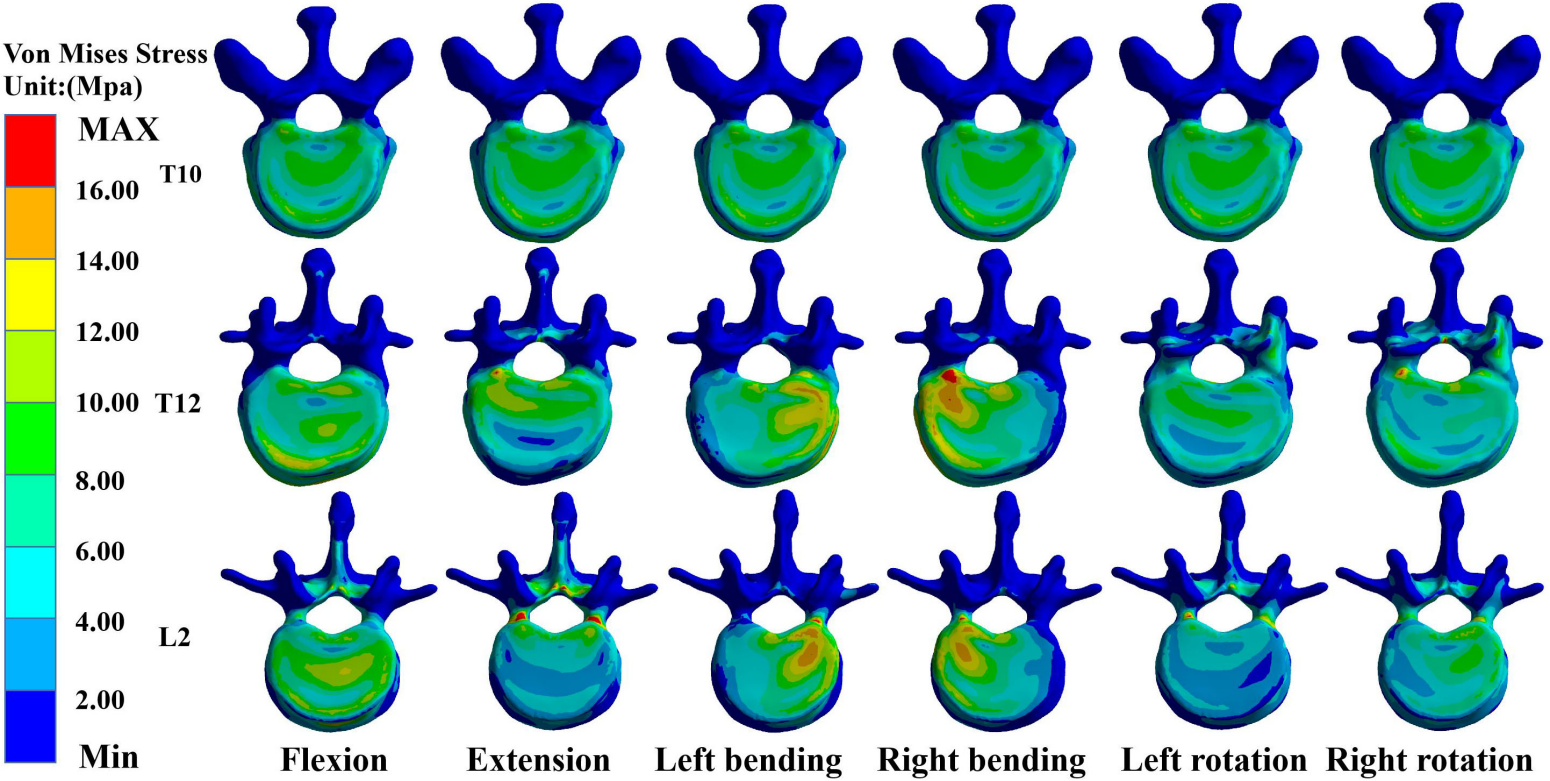
Maximum von Mises stress at T10, T12, and L2 in the M-T11 group.

In the M-T11 group, the maximum von Mises stress values were 19.70 MPa, 19.70 MPa, 19.71 MPa, 19.69 MPa, 20.26 MPa, and 19.71 MPa for T10 in the six directions of forward flexion, extension, left and right lateral bending, and left and right rotation, respectively, and 21.86 MPa, 21.81 MPa, 25.48 MPa, 27.42 MPa, 20.87 MPa, and 21.88 MPa for T12, respectively, while the maximum von Mises stress values were 20.84 MPa, 30.61 MPa, 26.15 MPa, 24.15 MPa, 19.54 MPa, and 17.65 MPa for L2 in the six directions (**Figure 4**).

### Maximum von Mises stress values for T10, T12, and L2 in M-T12

**Figure 5:**
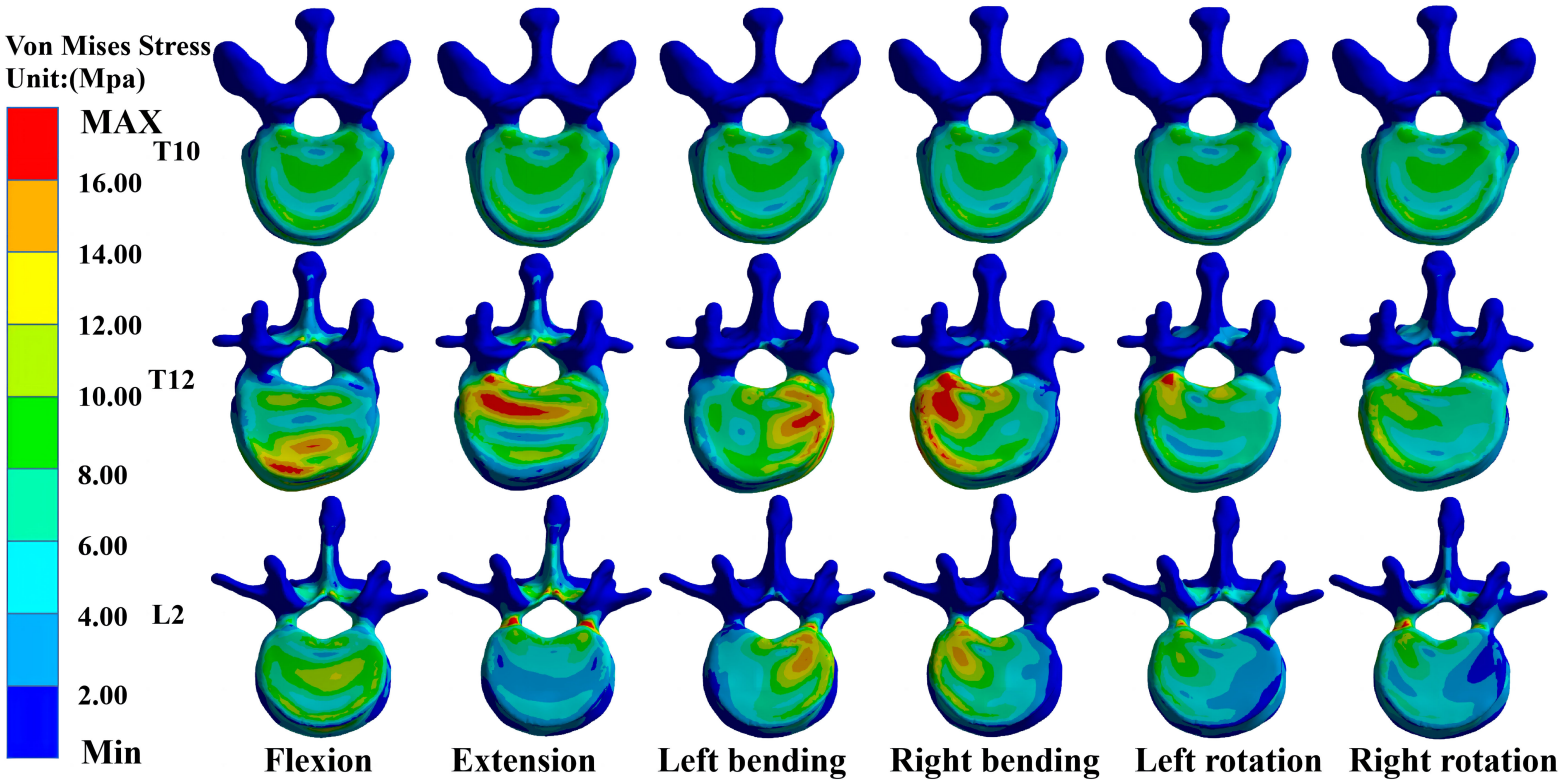
Maximum von Mises stress at T10, T12, and L2 in M-T12 group.

The maximum von Mises stress values for T12 were the greatest in the M-T12 group. The maximum von Mises stress values for T10 were 19.70 MPa, 19.70 MPa, 19.70 MPa, 19.0 MPa, 19.69 MPa, and 20.01 MPa in six directions of forward flexion, extension, left and right lateral bending, and left and right rotation, respectively, and 29.62 MPa, 32.63 MPa, 30.03 MPa, 31.25 MPa, 26.38 MPa, and 26.25 MPa for T12, respectively, and those for L2 were 20.84 MPa, 30.62 MPa, 26.17 MPa, 24.61 MPa, 22.62 MPa, and 22.82 MPa, respectively (**Figure 5**).

### Maximum von Mises stress values for T10, T12, and L2 in M-L1

**Figure 6:**
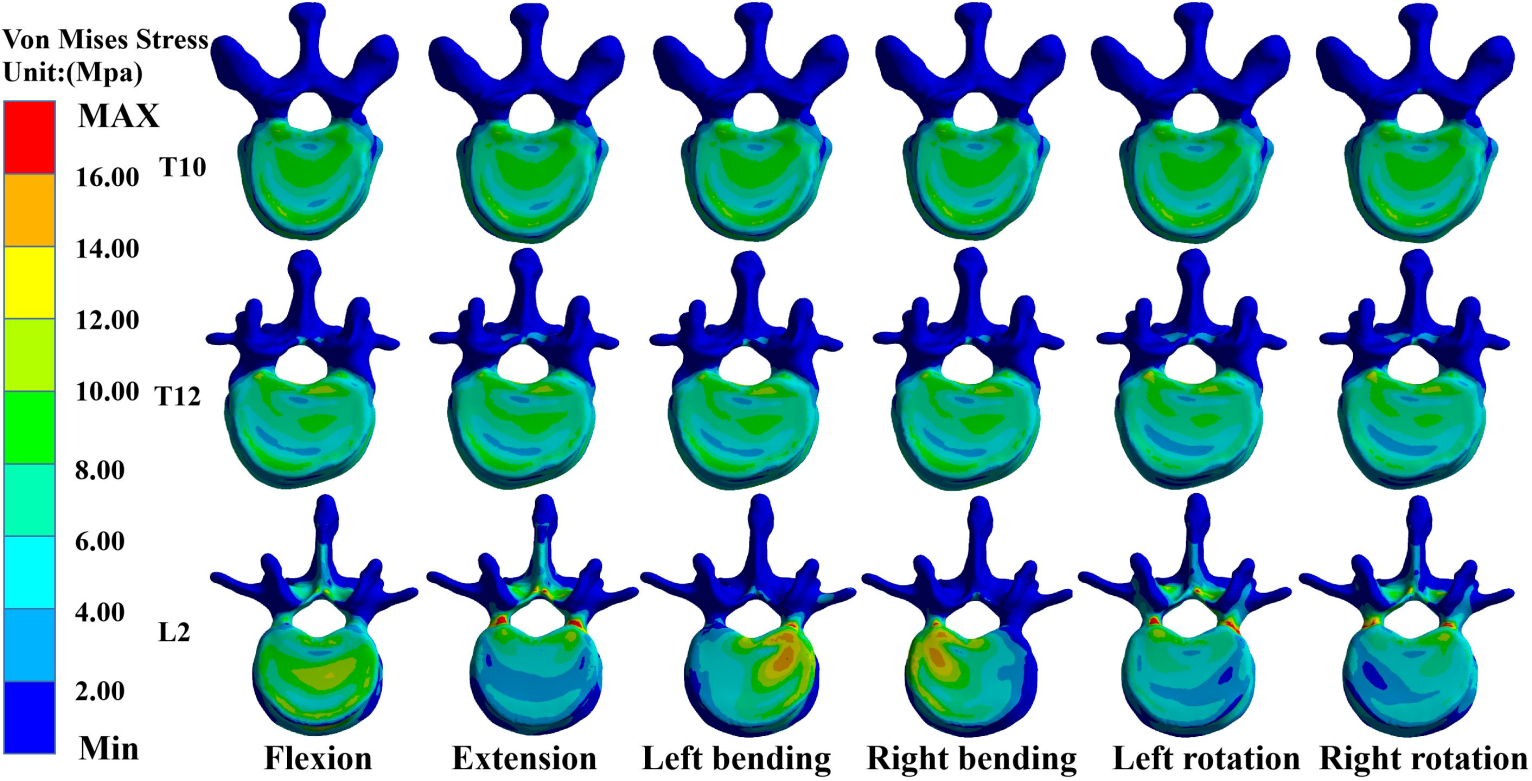
Maximum von Mises stress at T10, T12, and L2 in the M-L1 group.

The maximum von Mises stress values for L2 were the greatest in the M-L1 group. The maximum von Mises stress values of T10 were 19.70 MPa, 19.70 MPa, 19.70 MPa, 19.70 MPa, 20.27 MPa, and 20.27 MPa in six directions of forward flexion, extension, left and right lateral bending, and left and right rotation, respectively, while those for L2 were 19.37 MPa, 30.24 MPa, 26.19 MPa, 24.17 MPa, 25.5 MPa, and 27.51 MPa, respectively, and those for L2 were 20.37 MPa, 30.24 MPa, 26.19 MPa, 24.17 MPa, 25.55 MPa, and 27.51 MPa, respectively (**Figure 6**).

### Maximum von Mises stress values for T10, T12, and L2 in M-L2

**Figure 7:**
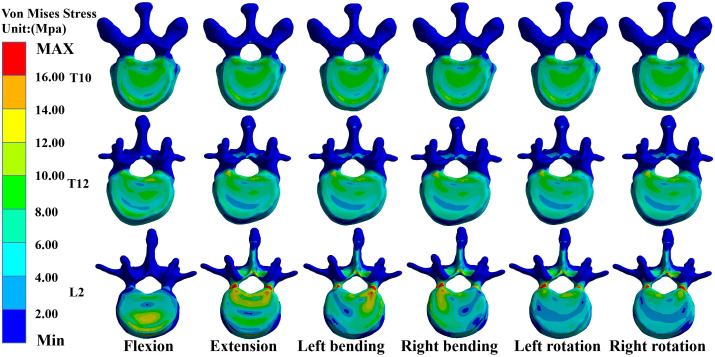
Maximum von Mises stress at T10, T12, and L2 in the M-L2 group.

**Figure 8:**
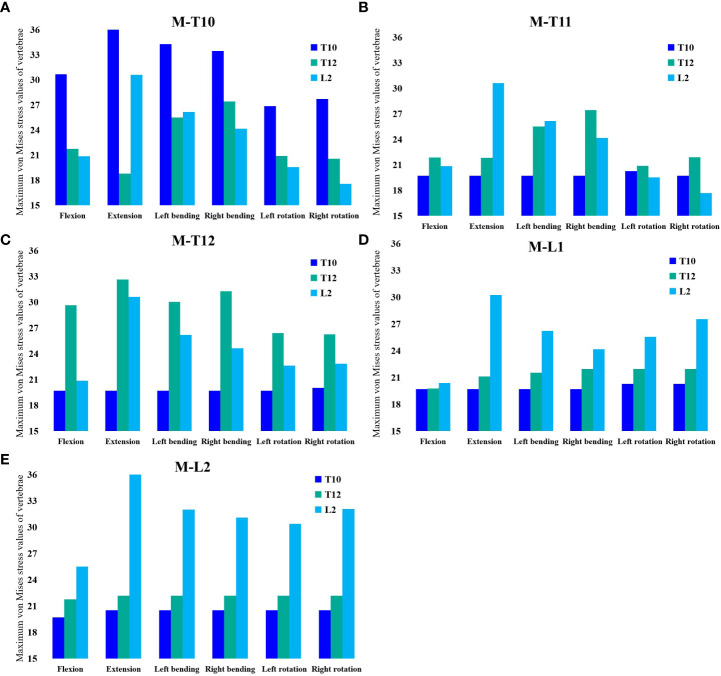
**(A)** Maximum von Mises stress histogram for T10, T12, and L2 in M-T10 group. **(B)** Maximum von Mises stress histogram for T10, T12, and L2 in M-T11 group. **(C)** Maximum von Mises stress histogram for T10, T12, and L2 in M-T12 group. **(D)** Maximum von Mises stress histogram for T10, T12, and L2 in M-L1 group. **(E)** Maximum von Mises histogram for T10, T12, and L2 in M-L2 group.

The maximum von Mises stress values for L2 were the greatest in the M-L1 group. The maximum von Mises stress values of T10 were 19.70 MPa, 20.52 MPa, 20.52 MPa, 20.52 MPa, 20.52 MPa, and 20.523 MPa in six directions of forward flexion, extension, left and right lateral bending, and left and right rotation, respectively, and the stresses for T12 were 21.74 MPa, 22.17 MPa, 22.17 MPa, 22.17 MPa, and 22.17 MPa in six directions, while the stresses for L2 were 25.48 MPa, 36.38 MPa, 31.99 MPa, 31.07 MPa, 30.36 MPa, and 32.07 MPa in six directions (**Figures 7**, **8**).

## Discussion

PVP is an effective treatment for osteoporotic vertebral compression fractures, relieving pain rapidly, reducing bed rest, restoring patients to their normal life in the shortest time, and improving their quality of life (13). However, an extensive literature review has shown that vertebral cement augmentation in these procedures increases the risk of subsequent new fractures and that the incidence of new fractures in adjacent vertebral bodies is higher than in non-adjacent vertebral bodies (23, 24). Few studies are available on this particular disease condition of the sandwich vertebra (25, 26). Although previous studies have hypothesized that the likelihood of vertebral sandwich fracture is high due to the bone cement double-load drift (27, 28), there is no biomechanical basis for comparing the risk of refracture between SVs and common adjacent vertebral bodies. Whether prophylactic injection of bone cement reinforcement should be performed in a sandwich vertebral body is inconclusive. This study investigates the biomechanical basis using the finite element method.

At present, the implementation of secondary fracture prevention worldwide is very unsatisfactory, and recent studies have emphasized the individualized management of patients after PVP surgery, especially the use of anti-osteoporosis drugs (29–31), We analyzed the stress of this special type of sandwich vertebral patients in daily life through mechanical experiments and explored and provided mechanical theoretical support for patients in the management of postoperative spinal posture. In our study, we found that T12 stress was not maximal in all groups except the M-T12, so the stress experienced by the sandwich vertebrae was not necessarily maximal during daily activities, depending on the degree of daily spinal curvature.

In our study, it was found that when the moment was at which vertebral segment, it withstood the greatest maximum von Mises stress. From this, it could be shown that when the spine moved, the stress point of the force emitted by various muscles directly acted on the certain vertebra at that time this vertebra withstood the greatest stress, which we called the stress target vertebra (STV), and the STV also changed with different degrees of spinal curvature. In our study, we set the upper two vertebrae of the sandwich vertebra, the sandwich vertebra itself, and the lower two vertebrae of the sandwich vertebra as the STV, and we observed that only when the sandwich vertebra became the STV did it withstand the greatest von Mises stress, at which point it became the most dangerous vertebra. When the STV was above the sandwich vertebra, the von Mises stress received by the sandwich vertebra at this time was also great because it was transmitted by the stress. As the stress target vertebra is closer to the sandwich vertebra, the stress received on the sandwich vertebra increases, whereas the stress on the sandwich vertebra is minimal when the stress target vertebra is located below the sandwich vertebra.

Previous studies have shown a potential relationship between new symptomatic fractures (NSFs) and enhancing vertebrae (23, 32). Jin Liu et al. (25) reported that the risk of NSF increases with vertebral type in clinical treatment in the following order: non-adjacent vertebrae < normal-adjacent vertebrae < sandwich vertebrae. Pu Jia (27) also suggested that sandwich vertebrae are more susceptible to fracture than adjacent vertebrae. However, Komemushi et al. (33) showed that SV was not a significant risk factor for developing new fractures. Wang et al. (28) compared the incidence of Sandwich vertebral fracture (9/42, 21.4%) with conventional adjacent level fractures (11/71%) in a clinical study, and the difference was not statistically significant (p = 0.424). In another study of 1,347 patients with vertebral compression fractures who underwent cement augmentation, Ping-Yeh Chiu (1) found that the incidence of conventional adjacent fractures was 16.4% (196/1,194), which did not differ significantly from the incidence of vertebral sandwich fractures (p = 0.188). Their study showed that vertebral sandwich segments were not associated with a higher fracture risk than adjacent segments. Dissecting vertebral fractures was not associated with an increased risk compared to fractures at adjacent levels.

Bo Yang et al. (9) collected the clinical data of 225 OVCF patients with sandwich vertebral bodies and common adjacent vertebral bodies to compare the incidence of postoperative fractures between sandwich vertebral bodies and common adjacent vertebral bodies. The incidence of sandwich vertebral fractures was comparable to that of adjacent vertebral bodies. Our study can explain why the results of these clinical studies on the incidence of sandwich vertebral fractures may differ. In addition to traumatic factors, patients vary in their long-term spinal motion patterns after surgery, and if patient habits or spinal degeneration make the spine present the same curved posture for a long time and increase the posture of specific spinal local stresses due to increased long-term local pressure, this vertebral body is prone to recurrent fractures because this may lead to insufficient blood supply, secondary minor bone damage, or bone loss (27).

Another question is whether prophylactic cement should be used to reinforce the sandwich vertebral body. Some studies have shown that prophylactic cement augmentation of adjacent vertebrae is beneficial (27, 34), while others have not (35). We believe that the sandwich vertebra may not be the most susceptible to secondary compression fractures, so prophylactic cement augmentation of the sandwich vertebra is unnecessary. We also believe that if the patient’s spinal activity after PVP causes the STV to be the reinforced vertebra or its upper adjacent vertebra, and bone cement increases the stiffness of the reinforced vertebral body, then the adjacent vertebra of the reinforced vertebral body is extremely dangerous if the patient is engaging in increased weight-bearing behavior.

## Conclusion

From our study, we found that patients who developed sandwich vertebrae after multiple PVP surgeries did not always have the greatest stress on the sandwich vertebrae during daily spinal activity, and the most stressed vertebrae would change continuously with the degree of spinal curvature. We should encourage patients to avoid the same spinal curvature posture for a long time in life and work or encourage patients to wear a spinal brace for protection after surgery because it may lead to a specific spine becoming an STV for a long time, bearing excessive load for a long time, and disrupting its blood supply, resulting in more severe loss of spinal quality and increasing the possibility of fractures.

Several limitations need to be discussed in our study: a) we simplified the tissue when creating the model, such as the characteristics of the disc, ligaments, and paravertebral muscles; these simplifications may have an impact on stress and displacement. The intervertebral disc is a fiber-reinforced porous elastic material (36), and its geometric features have a strong influence on its biomechanical behavior (37), Elmasry et al. (38) investigated thoracolumbar fractures, modeling the disc as a fibrotic-ally reinforced pore system, and focused on the effect of the transition zone between cement and cancellous bone on stress, making the disc and model more realistic (39). b) We refer to previous literature to simulate bone cement with vertical cylinders because it has been found that while different shapes of cement simulating PVP can produce different stresses and displacements, the same conclusion can be drawn, and the use of vertical cylinders to simulate bone cement not only can reach the same conclusion but also can reduce the computer calculation and ensure the repeatability of the study. c) Although our fractured vertebral model underwent planar cutting, it cannot represent the complexity of actual vertebral fractures and the diversity of vertebral morphology during the fracture process. d) Insufficient experimental control, future *in vitro* biomechanical experiments, and clinical studies should be conducted to evaluate the results of this study. e) The spinal model did not provide a detailed simulation of the microstructure of osteoporotic cancellous bone and was designated as 67% of the elastic modulus of healthy bone. f) We only investigated simple spinal bending and rotation movements. We have a basic understanding of the stress on the sandwich and other vertebral bodies during simple daily activities, and more sophisticated technical means are needed to simulate more complex activity scenarios in the future. g) This study did not include disc degeneration, and posterior instrumentation may be a good option if disc degeneration is severe (40, 41).

## Data availability statement

The raw data supporting the conclusions of this article will be made available by the authors, without undue reservation.

## Ethics statement

The studies involving humans were approved by This study involving human participants was reviewed and approved by the Ethics Committee of the Second Hospital of Xuzhou University ([2022] 070702). The studies were conducted in accordance with the local legislation and institutional requirements. The participants provided their written informed consent to participate in this study.

## Author contributions

SH: Conceptualization, Data curation, Formal Analysis, Investigation, Methodology, Project administration, Software, Supervision, Validation, Writing – original draft. LL: Conceptualization, Investigation, Software, Writing – original draft. CZ: Data curation, Methodology, Supervision, Writing – original draft. XZ: Formal Analysis, Project administration, Validation, Writing – original draft. ZT: Formal Analysis, Methodology, Supervision, Writing – original draft. XM: Formal Analysis, Investigation, Project administration, Writing – original draft. CX: Data curation, Project administration, Supervision, Writing – original draft, Funding acquisition, Resources, Writing – review & editing. XT: Funding acquisition, Methodology, Project administration, Resources, Visualization, Writing – original draft, Writing – review & editing.
